# *Ficus deltoidea* Jack: A Review on Its Phytochemical and Pharmacological Importance

**DOI:** 10.1155/2014/902734

**Published:** 2014-03-18

**Authors:** Hamidun Bunawan, Noriha Mat Amin, Siti Noraini Bunawan, Syarul Nataqain Baharum, Normah Mohd Noor

**Affiliations:** ^1^Institute of Systems Biology, Universiti Kebangsaan Malaysia, 43600 Bangi, Selangor, Malaysia; ^2^Biotechnology Research Centre, Malaysian Agricultural Research and Development Institute, P.O. Box 12301, 50774 Kuala Lumpur, Malaysia

## Abstract

*Ficus deltoidea* Jack (Moraceae) has had a long history of use in traditional medicine among the Malays to alleviate and heal ailments such as sores, wounds, and rheumatism and as an after-birth tonic and an antidiabetic drug. Modern pharmacological studies demonstrated that this plant has a wide variety of beneficial attributes for human health. Despite its importance, a review of this species has not been published in the scientific literature to date. Here, we review and summarize the historic and current literature concerning the botany, traditional uses, phytochemistry, pharmacological effects, and toxicity of this wonder plant. This summary could be beneficial for future research aiming to exploit the therapeutic potential of this useful, medicinal species.

## 1. Introduction

For many years there has been a tremendous interest in biological products gained from megadiversity countries like Malaysia. These invaluable products are explored for the potential discovery of novel biomolecules that have possible future uses. Recently, natural plant products have been gaining popularity in the prevention and treatment of various diseases. Traditional medicines from plants have attracted major attention worldwide because of their potential pharmaceutical importance.

One of the most popular and well-known plants with a long history of use among the Malays is* Ficus deltoidea *Jack, a plant of the family Moraceae.* Ficus deltoidea* not only has been used as a medicine for various ailments in the Malay Archipelago, but also has been distributed and formulated as capsules, tea, and tonic tea throughout Malaysia. This review will unify fragmented information regarding* F. deltoidea* in terms of its botany, traditional uses, phytochemistry, and pharmaceutical effects in order to facilitate better understanding and provide further support for the ethnopharmacological use of this important species.

## 2. Botany 

### 2.1. Botanical Name

Its botanical name is* Ficus deltoidea* Jack.

#### 2.1.1. Subsp.**** *Deltoidea*

The synonyms are* F*.* deltoidea* var.* angustifolia* (Miq.) Corner,* F*.* deltoidea* forma* angustissima* Corner,* F*.* deltoidea* var.* arenaria* Corner,* F*.* deltoidea* var.* bilobata* Corner,* F*.* deltoidea* var.* borneensis *Corner,* F*.* deltoidea* forma* subhirsuta* Corner,* F*.* deltoidea* var.* kunstleri* (King) Corner,* F*.* deltoidea* var.* lutescens* (Desf.) Corner,* F*.* deltoidea* forma* longipedunculata* Corner,* F*.* deltoidea* forma* subsessilis* (Miq.) Corner,* F*.* deltoidea* var.* peltata* Corner,* F*.* deltoidea* var.* recurvata* Kochummen, and* F*.* deltoidea* var.* trengganuensis* Corner [1].

#### 2.1.2. Subsp.* motleyana* (Miq.) C.C Berg, Comb. and Stat. Nov

The synonym is* F. deltoidea* var.* oligoneura* (Miq.) Corner [1].

### 2.2. Common Names

It is commonly known as mistletoe fig and also as Mas Cotek, Telinga Beruk, or Serapat Angin by Malays. Other vernacular names include Sempit-Sempit and Agoluran for people in Sabah, Sarawak, and the Kalimantan Islands, Tabat Barito in Indonesia, and Kangkalibang in Africa.

### 2.3. Botanical Description and Distribution

**Figure 1:**
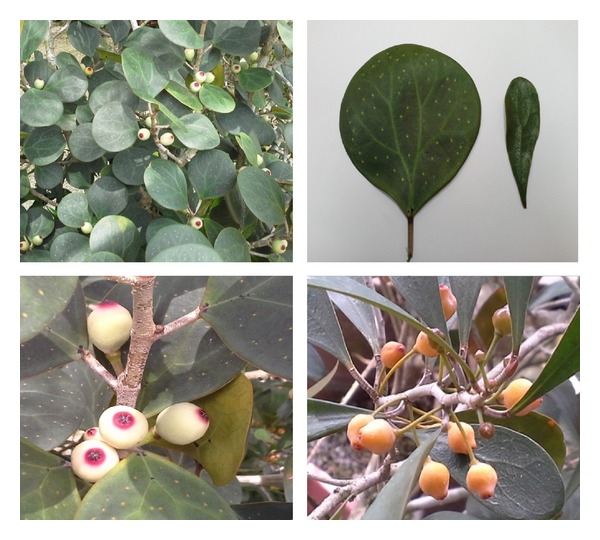
*Ficus deltoidea* commonly known as Mas Cotek in Malaysia.

*Ficus deltoidea* is an evergreen shrub that reaches a height of 2 meters, with whitish grey bark, leaves broadly spoon-shaped to obovate, with a leaf length between 4 cm and 8 cm, coloured bright green above, and rust-red to olive-brown beneath. The plant produces figs that are spherical to round, with a width of 1.0 to 1.5 cm and coloured yellow to orange-red (Figure 1). This plant is native to the Malayan Archipelago and has been introduced elsewhere. It is distributed throughout the region known as Malesia, which includes Thailand, Indonesia, and Malaysia [2].

## 3. Ethnobotanical Uses

Malays in the Peninsular Malaysia have been using the powdered root and leaves of* F. deltoidea* to treat wounds, rheumatism, sores, and other ailments for centuries. The decoction of boiled leaves of* F. deltoidea* is traditionally used as an antidiabetic treatment and an after-birth tonic to contract the uterus and vaginal muscles, to treat disorders of the menstrual cycle, and also to treat leucorrhoea [3]. Additionally,* F. deltoidea* fruits are traditionally chewed to relieve toothache, cold, and headache and the entire* Ficus deltoidea* plant is also traditionally used as an aphrodisiac tonic and as health tonic by women in Indonesia.

## 4. Chemical Composition

A wide range of chemical compounds have been isolated and characterized from* F. deltoidea*, particularly from the leaves and figs. An initial comprehensive study on volatile compounds produced by the fruits was conducted by Grison-Pigé et al. [4, 5]. The volatile compounds isolated and identified are mainly products of the shikimic acid pathway, terpenoids, and aliphatic groups, generally present as floral fragrances in plants. In 2009, Mohd Lip et al. [6] isolated and identified moretenol from* F. deltoidea* leaves using nuclear magnetic resonance (NMR) and mass spectrometers. Later on, an antibacterial compound known as lupeol (C_30_H_50_O) was also isolated from the leaves of* F. deltoidea* and exhibited toxicity against* Staphylococcus aureus*,* Bacillus subtilis, *and* Escherichia coli* [7]. Ong et al. [8] demonstrated enhancement of flavonoid compounds (rutin, quercetin, and naringenin) in cell cultures of* F. deltoidea* influenced by different carbon sources as well as plant growth regulators. A comprehensive study on flavonoid compounds of aqueous extract of* F. deltoidea* was conducted by Omar et al. [9] using HPLC-MS-based methodology and more than 25 compounds were identified. Currently, two bioactive constituents known as vitexin and isovitexin have been isolated, identified, and evaluated to show *α*-glucosidase inhibition [10]. The metabolites identified from* F. deltoidea* to date are presented in Table 1.

## 5. Pharmacological Properties

### 5.1. Antidiabetic

*Ficus deltoidea* is traditionally used in Malaysia to control blood sugar levels. Adam et al. [11] evaluated the potential of five extracts and three fractions of* F. deltoidea* in order to enhance basal and insulin-stimulated glucose uptake into the Chang liver cell line. The results show that* F. deltoidea* extracts and fractions, with the exception of petroleum ether, possess the potential to stimulate basal and insulin-stimulated glucose uptake into the liver cell line, with ethanolic extract having the highest insulin mimetic activity. Another study by Adam et al. [12] reported that hot aqueous, ethanolic, and methanolic extracts of* F. deltoidea* inhibit rat intestine *α*-glucosidase activity significantly, an enzyme that is accountable for carbohydrate digestion to condense the effect of carbohydrate in blood sugar. This was supported in an animal model in which all extracts reduced postprandial hyperglycemia after sucrose administration. Methanolic extract was found to be the most potent inhibitor of *α*-glucosidase [12].

Another study by Farsi et al. [13] reported that the n-butanol fraction from the leaves of* F. deltoidea* showed visible *α*-glucosidase and *α*-amylase inhibitory effects to control postprandial hyperglycemia. A different study conducted by Adam et al. [14] to evaluate the ethanolic effect of* F. deltoidea* on glucose levels in normal rats showed that all doses reduced fasting blood glucose, especially after 6 hours of administration. Adam et al. [15] also evaluated extracts of* F. deltoidea* for hyperglycemia effects at different prandial states. The study found that hot aqueous extracts of* F. deltoidea* stimulate insulin secretion and show a high magnitude of stimulation and the extract induced the usage of intracellular Ca^2+^ to initiate release of insulin, whilst increased basal and insulin-mediated glucose uptake into adipocytes cells was observed with ethanolic and methanolic extracts.

**Figure 2:**
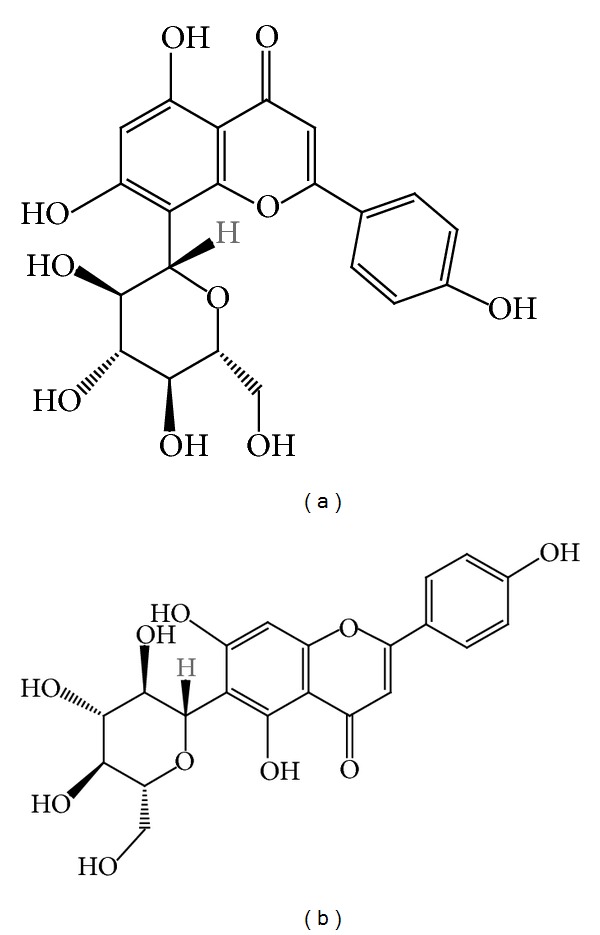
(a) Vitexin and (b) isovitexin chemical structures.

Partitioned extracts, subfractions, and pure bioactive constituents were the subject of an *α*-glucosidase inhibition assay. Both vitexin and isovitexin significantly reduced postprandial blood glucose level in mice, were nontoxic even at the highest dose of 2 g per kg, and exhibited* in vivo* *α*-glucosidase activity (Figure 2) [10]. Draman et al. [16] evaluated the effects of* F. deltoidea* leaves on fasting blood sugar and renal and lipid profiles of type 2 diabetic humans in Malaysia. The patients were given* F. deltoidea* 350 mg orally twice daily for two months. Even though the patients reported feeling energetic and fresh, effects on fasting blood sugar, HbA1C, and renal and lipid profiles were not significant. On the other hand, Kalman et al. [17] reported that* F. deltoidea* has significant effect on reducing glucose and lipid levels in human body after testing the antidiabetic effects of* F. deltoidea *in adults with prediabetes.

Recently, the fruits of two varieties of* F. deltoidea*, var.* angustifolia* and var.* kunstleri,* were separately extracted and the antidiabetic activities were evaluated according to the ability of the extract to inhibit both yeast and mammalian *α*-glucosidase and *α*-amylase [18]. The study also found that the crude extracts and fractions of both varieties hindered yeast and rat intestinal *α*-glucosidases depending on dose used but failed to inhibit porcine pancreatic *α*-amylase.

### 5.2. Anti-Inflammatory and Antinociceptive Activity

The anti-inflammatory activity of the aqueous extract of* F. deltoidea* leaf has been evaluated in rats using a carrageenan-induced paw edema test, a cotton pellet-induced granuloma test, and a formalin test, demonstrating that* F. deltoidea* had significant anti-inflammatory activity in every test with dose-response effects observed [19]. The leaves possess anti-inflammatory effects against various inflammatory responses as well as pain-associated inflammatory responses. Previously, Abdullah et al. [20] evaluated standardised extracts of different varieties of* F. deltoidea* for anti-inflammatory activity using three* in vitro* assays: lipoxygenase, hyaluronidase, and TPA-induced edema. The results demonstrated that extracts of leaves of* F. deltoidea* have significant anti-inflammatory properties, suggesting that the extracts may be useful for relieving pain by reducing inflammation.

Antinociceptive activity of* F. deltoidea* aqueous extract from leaves was studied by Sulaiman et al. [21] in order to verify using this plant to reduce sensitivity to painful stimuli using chemical and thermal nociception models. Three methods were used in their experiment: acetic acid-induced abdominal writhing, formalin, and a hot plate test. Significant antinociceptive effects of* F. deltoidea* leaves were observed in all three methods used when administered 30 minutes prior to pain induction. The results demonstrate that* F. deltoidea* leaves do have antinociceptive activity, corroborating its traditional use in treating painful conditions, such as headache and toothache.

### 5.3. Antimelanogenic and Antiphotoaging Effect

*Ficus deltoidea* was also reported to have antimelanogenic effects in reducing melanogenesis biosynthesis for melanin production [22].* Ficus deltoidea* extract activity was investigated using cultured B16F1 melanoma cells focusing on the characterization of tyrosinase, the enzyme that is required for melanin production. The extract from* F. deltoidea* inhibited mushroom tyrosinase activity and intracellular tyrosinase activity of B16F1. Microphthalmia-associated transcription factor (MITF) expression also decreased when* F. deltoidea* extract was applied, suggesting that* F. deltoidea* has strong potential for use as a depigmenting agent in cosmetics.

Treatment with* F. deltoidea* extract also significantly inhibited UV-induced TNF-*α*, IL-1*α*, IL-6, and COX-2 expression [23]. Decreased collagen synthesis of fibroblasts as a result of UVB exposure returned to normal after* F. deltoidea* extract treatment. Furthermore, following UVB irradiation enhanced MMP-1 expression that would usually be observed decreased after treatment with the* F. deltoidea* extract in a dose-dependent manner. The study concluded that the* F. deltoidea* extract may have a positive effect against UVB-induced skin damage and that it is useful for antiphotoaging cosmetic products [23].

### 5.4. Antioxidant Effect

**Figure 3:**
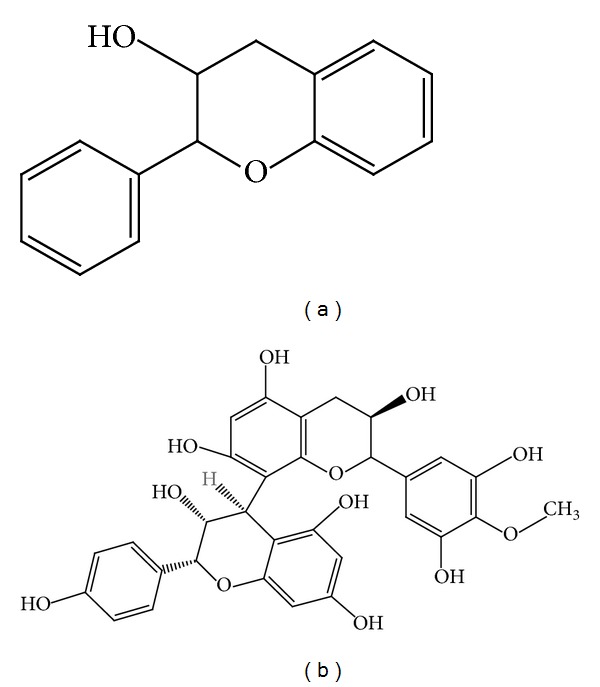
Chemical structure of (a) flavan-3-ol monomer and (b) proanthocyanidins.

The antioxidant property of the* F. deltoidea* extract was revealed through a total phenolic content and ferric reducing antioxidant potential (FRAP) assay by Omar et al. [9]. It was found that flavan-3-ol monomers and proanthocyanidins contributed 85% of the antioxidant activity of the aqueous extract of* F. deltoidea* (Figure 3). In contrast, this figure was 15% for flavones, primarily due to apigenin-6,8-C-diglucoside. Previously, Hakiman and Maziah [24] described another experiment where different aqueous extracts of* F. deltoidea* accessions were evaluated for their antioxidant activities using several assays such as FRAP, free radical scavenging assay, total polyphenol, flavonoid, phenolic acid, and vitamin C.

### 5.5. Antiulcerogenic Effect

The potential of* F. deltoidea* whole-plant extract as an ulcer healing agent for gastric ulcers caused by ethanol in Sprague Dawley rats was investigated by Zahra et al. [25]. Four groups of rats were used in the experiment: negative controls (treated with distilled water), 250 and 500 mg kg^−1^ of* F. deltoidea* extract, and positive controls (treated with omeprazole). Pretreatment with* F. deltoidea* extract demonstrated considerably less gastric mucosal lesions produced by ulcerogens compared to the negative control. Predictably, gastric protection was more effective with use of 500 mg kg^−1^ *F. deltoidea* extract compared to 250 mg kg^−1^. The negative control rats showed very severe gastric mucosal damage as well as edema and leucocyte permeation of the submucosal layer, suggesting that* F. deltoidea* extract protects against ulcers, demonstrated by the reduction in ulcer areas, inhibition of submucosal edema, and reduced leucocyte infiltration.

### 5.6. Wound Healing Activity

The effects of* F. deltoidea* extract for healing of wounds in Sprague Dawley rats were evaluated by Abdulla et al. [26]. The group that was treated with a placebo containing 5% and 10% of* F. deltoidea* extracts showed significantly accelerated wound healing in comparison with deionized water (negative control) and blank placebo treatments. The rats treated with* F. deltoidea* also showed decreased scar width, more fibroblast proliferation, and more collagen fibres accompanied by angiogenesis in the granulation tissue.

### 5.7. Antibacterial Activity

A study by Uyub et al. [27] found that* F. deltoidea *leaf extract has antimicrobial activity towards* Helicobacter pylori*, the major agent in chronic gastritis and gastric ulcers. Methanol extracts of* F. deltoidea* showed highest activity against* H. pylori* with an inhibition zone diameter of 12.0 ± 0.6 mm followed by petroleum ether 10.0 ± 0.6 mm and chloroform extract 8.0 ± 0.1 mm. Another study found that ethanol and methanol extracts of* F. deltoidea* leaves inhibited growth of* Bacillus subtilis *[28]. This was further supported by Samah et al. [29] who found methanol extracts of whole* F. deltoidea* exhibited antibacterial activity against* Staphylococcus aureus*,* Bacillus subtilis*,* Escherichia coli*, and* Pseudomonas aeruginosa* and antifungal activity towards* Candida albicans*.

### 5.8. Anticancer Effect

Aqueous and ethanol extracts of* F. deltoidea* were found to have anticancer activity against human ovarian carcinoma cell line A2780 using a cell-based assay and confirmed by microscopic observation [30]. The plant extracts gave IC_50_ value of 224.39 ± 6.24 *μ*g/mL and 143.03 ± 20.21 *μ*g/mL in aqueous and ethanolic extracts, respectively. Both of the plant extracts have different effects on cell growth: the aqueous extract promoted cell detachment, while the ethanolic extract decreased cell proliferation. This might be due to different types of phytochemical property in those two extracts. The study also reported that apoptosis was caused by both extracts at 1000 *μ*g/mL.

### 5.9. Miscellaneous Activity

A study was conducted to assess whether the aqueous and ethanolic extracts of* F. deltoidea* leaves possessed antithrombotic activity and to observe the effect of the extracts on sperm quality and testosterone levels in diabetic rats [31]. These rats were administered alloxan monohydrate, which is known to cause an increase in blood glucose level, blood clotting rate, and sperm abnormalities and a decrease in testosterone level. Interestingly, rats given aqueous and ethanolic leaf extracts of* F. deltoidea* demonstrated significantly increased testosterone levels, sperm count, and motility and showed a reduction in the blood clotting levels and blood glucose level, as well as a reduction in sperm abnormalities.

Samsulrizal et al. [32] conducted another similar study with the same result and they additionally considered lactate dehydrogenase C4 (LDH-C4), a specific isoenzyme in mammalian testes that plays a role in sperm capacitation. The effect of* F. deltoidea* on LDH-C4 activity was also seen positive for maintaining healthy sperm. A further study on the effects of* F. deltoidea* methanolic extract on the reproductive systems of male rats was conducted by Norrizah et al. [33]. Surprisingly, the study found that the leaf extract decreased testes and epididymis weight, sperm count, and sperm viability. Rats treated with stem extract showed a significant increase in epididymis weight and sperm count and viability, although reduction in testes weight and the number of normal sperm morphology was observed.

## 6. Cytotoxicity Activity

Comprehensive attempts have been made on efficiency and safety studies on* F. deltoidea* [34–36]. A study by Shafaei et al. [35] confirmed that* F. deltoidea* leaves do not contain toxic elements by evaluating toxicological elements (lead, cadmium, arsenic, and mercury) using atomic absorption spectroscopy technique, standardisation parameters (moisture, volatile, total ash, and acid insoluble ash), and Microbial Limit Test (MLT) for microbial contamination.

A genotoxicity study was performed using the Ames test with the TA98 and TA100* Salmonella typhimurium* strains and showed the* F. deltoidea* extract did not have any potential to induce mutations in the presence or absence of S9 metabolic activation [36]. Further study on the acute toxicity showed that the LD_50_ of the extract was greater than 5000 mg/kg on Sprague Dawley rats treated with five different doses of the extract. In the subchronic study, there were no significant adverse effects on body weight, organ weight, food consumption, mortality, haematology, histopathology, and clinical chemistry, except an increase in the serum urea level [36].

## 7. Conclusions 

The scientific research on* F. deltoidea* indicated that this plant has received increasing interest in recent years.* Ficus deltoidea *has been reported to have beneficial pharmaceutical uses as an antidiabetic, anti-inflammatory, antinociceptive, antimelanogenic, antiphotoaging, antioxidant, antiulcerogenic, and antibacterial agent. Phytochemical and pharmaceutical studies have validated the traditional uses of* F. deltoidea*; however, there is a need to investigate the chemical or bioactive components that are responsible for providing the specific properties. The principle bioactive metabolites have to be examined for their bioactivities and their mechanism of action needs to be determined, in conjunction with analysing the pharmacokinetics and physiological pathways of specific compounds in* F. deltoidea*. These may help to strengthen our understanding of this highly therapeutic plant for commercial exploitation.

## Figures and Tables

**Table 1:** Chemical constituents identified in *F. deltoidea*.

Plant part used	Compound	Class	References
Fig	4-Methylbenzaldehyde	Shikimic acid	[4]
	Methyl benzoate	Shikimic acid	[4]
	Indole	Shikimic acid	[4]
	Decanal	Aliphatics	[4]
	6-Methyl-5-hepten-2-one	Acyclic monoterpenes	[4]
	Myrcene	Acyclic monoterpenes	[4]
	(Z)-*β*-Ocimene	Acyclic monoterpenes	[4]
	(E)-*β*-Ocimene	Acyclic monoterpenes	[4]
	*cis*-Furanoid linalool oxide	Acyclic monoterpenes	[4]
	*trans*-Furanoid linalool oxide	Acyclic monoterpenes	[4, 5]
	Linalool	Acyclic monoterpenes	[4, 5]
	*cis*-Pyranoid linalool oxide	Acyclic monoterpenes	[4]
	*trans*-Pyranoid linalool oxide	Acyclic monoterpenes	[4]
	Hotrienol	Acyclic monoterpenes	[4]
	Perillene	Acyclic monoterpenes	[4]
	Limonene	Cyclic monoterpenes	[4]
	Dendrolasine	Sesquiterpenes	[4]
	*α*-Cubebene	Sesquiterpenes	[4]
	Cyclosativene	Sesquiterpenes	[4]
	*Α*-Ylangene	Sesquiterpenes	[4]
	*α*-Copaene	Sesquiterpenes	[4, 5]
	*β*-Bourbonene	Sesquiterpenes	[4]
	1,5-Diepi-*β*-bourbonene	Sesquiterpenes	[4]
	*β*-Cubebene	Sesquiterpenes	[4, 5]
	*β*-Elemene	Sesquiterpenes	[4]
	*α*-Gurjunene	Sesquiterpenes	[4]
	*α*-*cis*-Bergamotene	Sesquiterpenes	[4]
	*β*-Caryophyllene	Sesquiterpenes	[4, 5]
	*α*-Santalene	Sesquiterpenes	[4]
	Selina-3-6-diene	Sesquiterpenes	[4]
	*α*-*trans*-Bergamotene	Sesquiterpenes	[4, 5]
	*α*-Humulene	Sesquiterpenes	[4, 5]
	Alloaromadendrene	Sesquiterpenes	[4, 5]
	Aciphyllene	Sesquiterpenes	[4]
	Germacrene D	Sesquiterpenes	[4, 5]
	*β*-Selinene	Sesquiterpenes	[4]
	*α*d-Selinene	Sesquiterpenes	[4]
	*α*-Selinene	Sesquiterpenes	[4]
	Bicyclogermacrene	Sesquiterpenes	[4]
	*α*-Muurolene	Sesquiterpenes	[4]
	Germacrene A	Sesquiterpenes	[4, 5]
	*δ*-Amorphene	Sesquiterpenes	[4]
	(E,E) *α*-Farnesene	Sesquiterpenes	[4, 5]
	2-*epi*-*α*-Selinene	Sesquiterpenes	[4]
	*δ*-Cadinene	Sesquiterpenes	[4, 5]
	Cadina-1,4-diene	Sesquiterpenes	[4]
	Germacrene B	Sesquiterpenes	[4]
	Caryophyllene oxide	Sesquiterpenes	[4]

Leaves	Gallocatechin	Flavonoids	[9]
	Epigallocatechin	Flavonoids	[9]
	Catechin	Flavonoids	[9]
	(Epi)afzelechin-(epi)catechin	Flavonoids	[9]
	(Epi)afzelechin-(epi)afzelechin	Flavonoids	[9]
	(Epi)catechin Epicatechin	Flavonoids	[9]
	Luteolin-6,8-C-diglucoside (lucenin-2)	Flavonoids	[9]
	Apigenin-6,8-C-diglucoside (vicenin-2)	Flavonoids	[9]
	Luteolin-6-C-hexosyl-8-C-pentoside	Flavonoids	[9]
	Luteolin-6-C-glucosyl-8-C-arabinoside	Flavonoids	[9]
	Apigenin-6-C-arabinosyl-8-C-glucoside (isoschaftoside)	Flavonoids	[9]
	Luteolin-6-C-arabinosyl-8-C-glucoside	Flavonoids	[9]
	Apigenin-6-C-glucoside-8-C-arabinoside (schaftoside)	Flavonoids	[9]
	Luteolin-8-C-glucoside (orientin)	Flavonoids	[9]
	Apigenin-6-C-pentosyl-8-C-glucoside	Flavonoids	[9]
	Apigenin-8-C-glucoside (vitexin)	Flavonoids	[9, 10]
	Apigenin-6-C-glucosyl-8-C-pentoside	Flavonoids	[9]
	Apigenin-6,8-C-dipentoside isomer	Flavonoids	[9]
	Apigenin-6-C-glucoside (isovitexin)	Flavonoids	[9, 10]
	4-p-coumarolyquinic acid	Flavonoids	[9]
	Moretenol	Terpenes	[6]
	(3*β*,13*ξ*)-Lup-20(29)-en-3-ol (lupeol)	Triterpenes	[7]

Cell culture	Rutin	Flavonoids	[8]
	Quercetin	Flavonoids	[8]
	Naringenin	Flavonoids	[8]
